# Carbon Nanotube/Poly(dimethylsiloxane) Composite Materials to Reduce Bacterial Adhesion

**DOI:** 10.3390/antibiotics9080434

**Published:** 2020-07-22

**Authors:** Márcia R. Vagos, Marisa Gomes, Joana M. R. Moreira, Olívia S. G. P. Soares, Manuel F. R. Pereira, Filipe J. Mergulhão

**Affiliations:** 1 LEPABE—Laboratory for Process Engineering, Environment, Biotechnology and Energy, Faculty of Engineering, University of Porto, Rua Roberto Frias, 4200-465 Porto, Portugal; marciavagos@gmail.com (M.R.V.); marisagomes@fe.up.pt (M.G.); joanarm@fe.up.pt (J.M.R.M.); 2 LCM—Laboratory of Catalysis and Materials, Associate Laboratory LSRE/LCM, Department of Chemical Engineering, Faculty of Engineering, University of Porto, Rua Roberto Frias s/n, 4200-465 Porto, Portugal; salome.soares@fe.up.pt

**Keywords:** carbon nanotubes, poly(dimethylsiloxane), adhesion, *Escherichia coli*

## Abstract

Different studies have shown that the incorporation of carbon nanotubes (CNTs) into poly(dimethylsiloxane) (PDMS) enables the production of composite materials with enhanced properties, which can find important applications in the biomedical field. In the present work, CNT/PDMS composite materials have been prepared to evaluate the effects of pristine and chemically functionalized CNT incorporation into PDMS on the composite’s thermal, electrical, and surface properties on bacterial adhesion in dynamic conditions. Initial bacterial adhesion was studied using a parallel-plate flow chamber assay performed in conditions prevailing in urinary tract devices (catheters and stents) using *Escherichia coli* as a model organism and PDMS as a control due to its relevance in these applications. The results indicated that the introduction of the CNTs in the PDMS matrix yielded, in general, less bacterial adhesion than the PDMS alone and that the reduction could be dependent on the surface chemistry of CNTs, with less adhesion obtained on the composites with pristine rather than functionalized CNTs. It was also shown CNT pre-treatment and incorporation by different methods affected the electrical properties of the composites when compared to PDMS. Composites enabling a 60% reduction in cell adhesion were obtained by CNT treatment by ball-milling, whereas an increase in electrical conductivity of seven orders of magnitude was obtained after solvent-mediated incorporation. The results suggest even at low CNT loading values (1%), these treatments may be beneficial for the production of CNT composites with application in biomedical devices for the urinary tract and for other applications where electrical conductance is required.

## 1. Introduction

The recent advancements in carbon nanotube (CNT) science have opened up promising possibilities for the development of novel materials and devices in the biomedical field [1]. Their nano-dimensional structure allied to a unique set of properties, such as a large aspect ratio, high surface energy density, electrical and thermal conductivity, as well as superior mechanical strength, flexibility, and ability to blend with other materials to form nanocomposites, have prompted their integration in biomaterials with enhanced properties [2,3,4].

In the last decade, CNT-polymer nanocomposites have been extensively used in pharmaceutical and medical fields, often showing remarkable improvements in the mechanical, electrical, optical, thermal, and structural properties of the composite material in relation to the polymer alone [2]. Due to their vast potential in the biomedical nanotechnology field, CNTs have been used not only in the construction of biosensors for the detection of biomolecules and cells but also in the development of drug delivery systems [1,5,6].

Studies on the direct cell-CNT interactions have provided indications that nanotubes have the potential to strongly adhere to cell membranes, so there has been an interest in the use of CNTs as coatings for cell culture substrates or medical implants to increase cell attachment and growth [7,8,9]. Likewise, CNT incorporation into polymers has shown to enhance cell attachment and proliferation, with beneficial implications in cell culture substrates and tissue engineering scaffolds [10,11,12,13,14,15,16,17]. However, as far as implantable medical devices are concerned, microbial adhesion on the implant surface often results in severe infections and failure of the implant. Therefore, contrary to having surfaces with enhanced cell attachment capability, developing surfaces capable of reducing bacterial adhesion becomes a major concern. The antibiofouling properties of CNTs, which are mainly related to their resistance against protein adhesion and other fouling components, also make them an attractive nanomaterial for a wide range of applications [4]. Their antimicrobial activity mainly depends on their length, physical disposition (degree of entanglement), and number of layers [18]. Different studies have indeed reported the efficacy of multiwalled carbon nanotube (MWCNT)/polymer composites in the reduction of bacterial adhesion and biofilm formation [19,20,21,22]. Modification of the surface of CNTs has also proved to play an important role in their antifouling potential [10,23].

In the biomedical industry, poly(dimethylsiloxane) (PDMS) has been widely used in the fabrication of medical devices and implants [24]. Particularly in the fabrication of urinary tract devices, PDMS is often used due to its high biocompatibility, mechanical resistance, and good chemical stability [25,26]. Despite these excellent properties, as with any other silicone material, PDMS is prone to non-specific surface adsorption of proteins and bacteria, which can be a disadvantage in the biomedical field. Recently, different studies have reported the increase of the antifouling properties of PDMS by the incorporation of MWCNTs [27,28,29,30].

Regarding CNT/PDMS composites, several reports have been published on their mechanical, electrical, and thermal properties, showing that the CNT incorporation can be beneficial [3,31,32,33,34,35,36,37,38]. Another example of the application of these composites is in the fabrication of electrically conductive materials for implants with sensing capacity [39,40,41,42,43]. Therefore, a deeper investigation of the interactions between bacterial cells and CNT-polymer composites at the interface level could provide insights into the use of CNT-based coatings in medical implants. Testing of these new composites should be performed in hydrodynamic conditions that are relevant for their final application [44] as flow affects the transport of bacteria to the surface and also the forces that adhered cells have to withstand [45]. Additionally, it has been shown that initial adhesion can provide very useful information regarding the antifouling performance of a surface or a coating [46,47].

The present study aimed at evaluating the bacterial adhesion behavior onto CNT/PDMS composite coatings. In order to assess the influence of CNT surface chemistry and dispersion state on cell adhesion, pristine, functionalized, and ball-milled CNTs were used. The materials were characterized in terms of thermal stability, surface hydrophobicity and morphology, and direct current (DC) electrical conductivity. Bacterial adhesion assays were performed in a parallel-plate flow chamber (PPFC) system using *Escherichia coli* as model bacteria.

## 2. Results and Discussion

### 2.1. Materials Characterization

The values of Brunauer–Emmett–Teller surface area (S_BET_) obtained for the pristine-CNT (p-CNT), functionalized-CNT (f-CNT), ball-milled p-CNT (p-BM) and ball-milled f-CNT (f-BM) samples were 289 m^2^ g^−1^, 361 m^2^ g^−1^, 375 m^2^ g^−1^ and 348 m^2^ g^−1^, respectively. These values indicate that the four samples of CNTs have different textural properties due to structural changes on f-CNT induced during the nitric acid treatment and also due to the milling process. It has been shown that acidic functionalization increases structural defects of the CNTs and opens their end caps [48,49], increasing the available area for adsorption [50]. The ball-milling treatment breaks the tubes, being responsible for the increase in the surface area observed for sample p-BM [51]. In the case of the sample f-BM, the surface area remains almost unchanged, probably due to the lower ball-milling treatment time applied to this sample and due to a higher agglomeration of the material (induced by the oxygen-containing surface groups and the ball-milling). The different textural properties of the two types of CNTs used may affect the bonding strength between the nanotubes and the PDMS chains.

**Figure 1:**
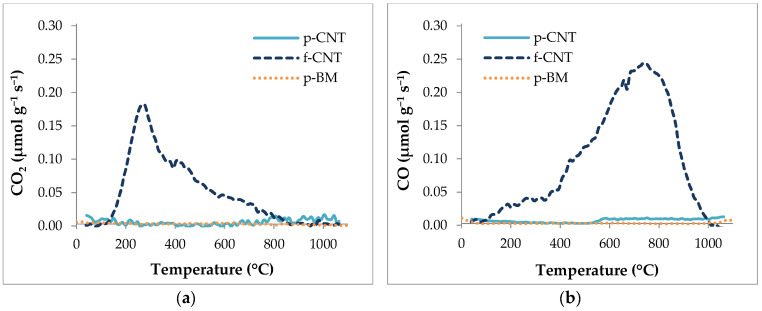
Temperature programmed desorption results of CO_2_ spectra (**a**) and CO spectra (**b**) of carbon nanotube (CNT) samples: pristine-CNT (p-CNT), functionalized-CNT (f-CNT) and ball-milled p-CNT (p-BM).

Temperature programmed desorption (TPD) analysis was performed to assess the content of functional groups introduced on the surface of the CNTs by the acid treatment and compare it to p-CNT. In this analysis, the amount of CO and CO_2_ released from the CNT sample reflects the type of oxygenated groups present at their surfaces, which in turn depends on the applied treatment. Results are shown in Figure 1, where it can be seen that f-CNT contained a large amount of oxygenated surface groups, which corroborates the acidic character of this sample when compared to p-CNT.

In fact, the surface of CNTs after functionalization with HNO_3_ contains a large amount of carboxylic acid groups (released as CO_2_ below 400 °C), lactones (released as CO_2_ around 650 °C) and some carboxylic anhydrides (released as CO and CO_2_ at around 550 °C). Phenol groups (released as CO at around 700 °C) and carbonyl/quinone groups (released as CO at around 850 °C) are also present [52,53]. The ball-milling treatment does not change the chemical surface of the CNTs [51].

**Figure 2:**
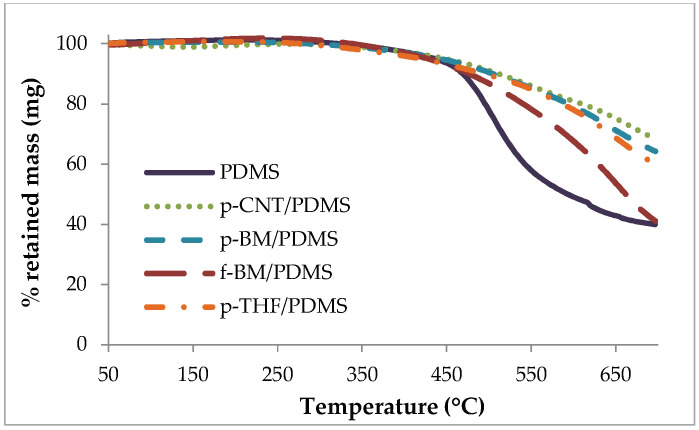
Thermogravimetric spectra of poly(dimethylsiloxane) (PDMS), p-CNT/PDMS, p-BM/PDMS, ball-milled f-CNT (f-BM)/PDMS and THF-treated p-CNT (p-THF)/PDMS composites.

The results of the thermogravimetric analysis (TGA) are shown in Figure 2. In the TGA spectra, it can be seen that PDMS started to pyrolyze at 450 °C, with a rapid mass loss until 600 °C. The composite samples showed a very different thermal behavior, with a smooth decomposition resulting in a lower percentage of mass loss. The p-CNT/PDMS sample showed the highest stability, suggesting that the interfacial bonding with PDMS was stronger for this material. Furthermore, it seems that the texture of the CNTs is not a critical factor in the thermal stability of the composite (p-BM/PDMS curve). However, with f-BM/PDMS, there was a significant difference in the decomposition, showing a higher weight loss compared to the other composites. This seems to indicate that the surface chemistry of the CNTs plays a more important role in the filler-polymer interactions than their structure since the oxygen-containing groups of the functionalized CNTs introduce polar moieties in the matrix, which have low affinity for the PDMS chains due to sterical and electrostatic repulsions [54,55,56,57].

Interestingly, the addition of tetrahydrophuran (THF) in the fabrication of the composites through the solution mixing method did not seem to have a negative impact on the polymer-CNT bondings, as can be seen from the TGA curve of THF-treated p-CNT (p-THF)/PDMS, which is very similar to that of p-BM/PDMS, suggesting that THF, even after being removed from the composite, served as an interfacial agent with PDMS. In general, from these results, it can be concluded that the interactions between the PDMS matrix and the CNTs are effective, resulting in an enhancement of the thermal stability of all the composites.

**Figure 3:**
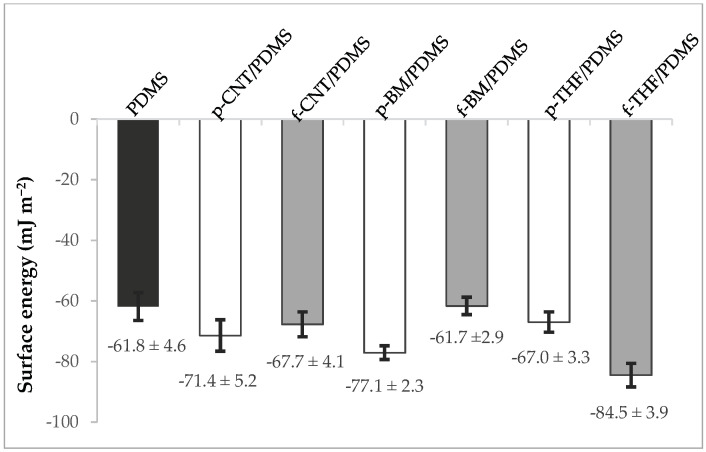
Surface free energy and adhesion energy of PDMS (black bar), p-CNT/PDMS, p-BM/PDMS and p-THF/PDMS composites (white bars); and f-CNT/PDMS, f-BM/PDMS and THF-treated f-CNT (f-THF)/PDMS composites (gray bars).

The results of the contact angle (CA) measurements are shown in Figure 3 and Table 1. All the surfaces produced were hydrophobic (negative ΔG_s_^TOT^ value). In addition, in all the surfaces, the acid-base forces had a greater contribution to the overall surface energy than the Lifshitz–van der Waals’ forces, showing the importance of the electron donor forces. The results showed that the p-CNT/PDMS and p-BM/PDMS surfaces were more hydrophobic than the f-CNT/PDMS and f-BM/PDMS surfaces, respectively (*p* < 0.05). Surprisingly, the p-THF/PDMS was found to be less hydrophobic than the THF-treated f-CNT (f-THF)/PDMS surface (*p* < 0.05), which was the most hydrophobic one. The surface with the least hydrophobic character was f-BM/PDMS, and all the other surfaces were more hydrophobic than PDMS (*p* < 0.05).

These results are in agreement with a previous study carried out by Beigbeder and its co-workers, where MWCNTs/PDMS composites showed a higher hydrophobicity when compared with unfilled PMDS [30]. It should be noted that these two groups of surfaces with opposing tendencies were also prepared using two different methods—bulk mixing and solution mixing. The surfaces produced by the bulk mixing process were more hydrophobic with p-CNT then with f-CNT, which can be explained because p-CNT is very hydrophobic, thus raising the overall hydrophobicity of the composite, whereas f-CNT has an acidic character due to the oxygenated surface groups, therefore yielding less hydrophobic composites [58]. However, the p-THF/PDMS and f-THF/PDMS composites showed an opposite tendency. It has been shown that THF can easily disperse CNTs [59], with reportedly improved results for functionalized CNTs [35]. However, in the present work, no comparative assessment was made on the dispersion of the two composites, so this aspect requires further analysis.

**Figure 4:**
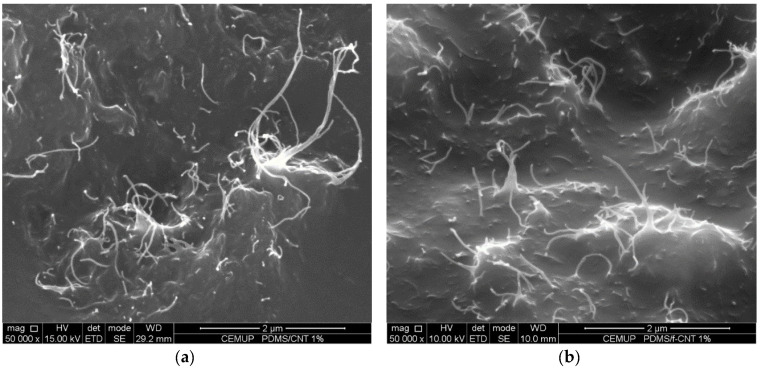

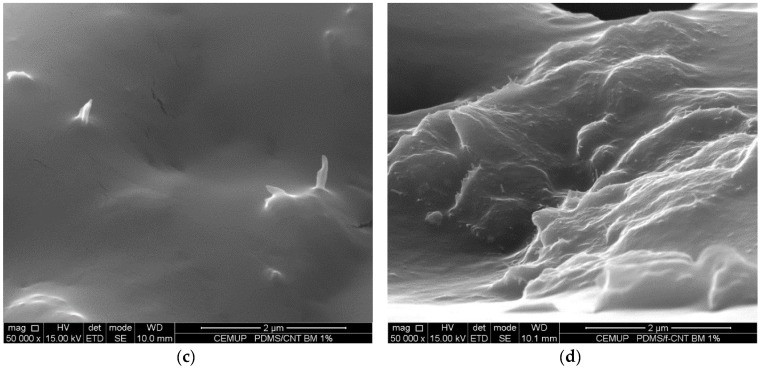
Scanning electron microscopy images of samples’ surfaces (magnification 50,000×); (**a**) p-CNT/PDMS; (**b**) f-CNT/PDMS; (**c**) p-BM/PDMS; and (**d**) f-BM/PDMS.

In order to assess the effect of the ball-milling procedure, Scanning Electron Microscopy (SEM) analysis of the surface of the p-CNT/PDMS, f-CNT/PDMS, p-BM/PDMS, and f-BM/PDMS composite samples was performed (Figure 4). The images show some sections of the composite surfaces where the CNTs were more densely packed. It can be seen in these regions that the CNTs formed small elevations and were closer to the surface. The p-CNT, f-CNT, p-BM, and f-BM samples all presented a similar morphological aspect; however, a decrease in the length of the MWCNTs is noticed as a result of the ball-milling treatment. It has been previously shown that ball-milling cuts the tubes (decreasing their lengths) and promotes their disentanglement [51].

Electrical measurements were performed to evaluate the influence of the different MWCNT surface treatments in the overall electrical response of the composites (Table 2). Despite the excellent electrical properties of CNTs, and their role in the enhancement of the final conductivity of a polymer composite [60,61], the present results revealed that the composites produced by the bulk mixing process (p-CNT/PDMS, f-CNT/PDMS, p-BM/PDMS, and f-BM/PDMS) did not exhibit any increase in electrical conductivity, as can be seen from the very low conductance values, which were all of the same order of magnitude as that of PDMS. This fact indicates that the CNTs were not well-enough dispersed in these samples to establish a percolative path for the current to flow with the CNT concentrations used.

However, the f-THF and p-THF samples showed a significant increase in electric conductance by five to seven orders of magnitude, respectively, which indicates that even at CNT concentrations as low as 1%, there is an enhancement in conduction driven by their better dispersion in PDMS, which made possible the formation of effective conduction paths along the nanotubes. In fact, the use of an adequate solvent to wet CNTs has already proven its worth in the improvement of the electrical properties of the CNT/PDMS composites [62]. Note that the top micrometer PDMS layer covering the CNTs, which was thicker in the case of the coatings fabricated by bulk mixing, may have acted as an insulating layer, hampering the measurements of current flowing through them, which could also explain why conductance values of different orders of magnitude were obtained.

A tendency for higher values of conductance of the composites prepared with non-functionalized CNTs (p-CNT/PDMS and p-THF/PDMS), compared with the functionalized ones, was observed. These results are supported by previous studies that have shown an electrical conductivity of p-CNT/polymer composites several orders higher than that of carboxylic f-CNT/polymer composites [63,64]. In the case of the samples treated with THF, this increase is even more significant. In fact, values obtained with p-THF/PDMS were nearly two orders of magnitude larger than those obtained with f-THF/PDMS. According to Carabineiro, et al. [65], as π orbitals are responsible for CNTs conductance [66], the lower conductivity obtained for the composites with f-CNT can be explained by the defects caused on the π orbitals during the functionalization process.

### 2.2. E. coli Adhesion Assays

**Figure 5:**
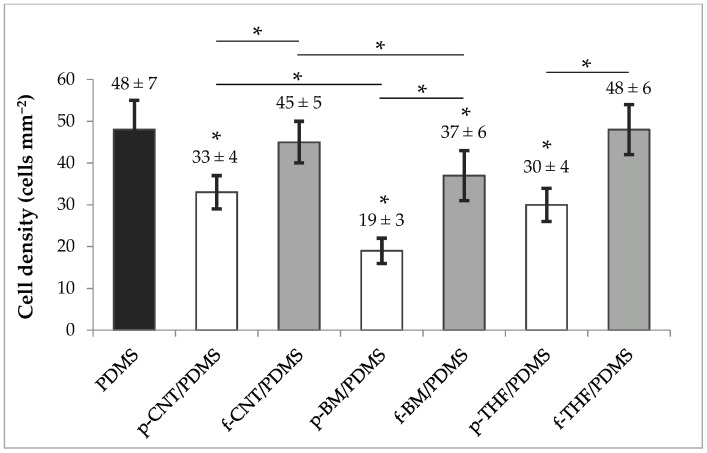
Bar chart of the average cell densities of PDMS (black bar), p-CNT/PDMS, p-BM/PDMS, and p-THF/PDMS (white bars); and f-CNT/PDMS, f-BM/PDMS, and f-THF/PDMS (gray bars) coatings, obtained with three independent tests. Error bars correspond to standard deviation. * indicates *p* < 0.0001, and consequently statistically different from the control.

The results of the bacterial adhesion assays with *E. coli* are presented in Figure 5. The bars correspond to the cell density after a 30 min adhesion assay. Results show that p-CNT/PDMS, p-BM/PDMS, f-BM/PDMS, and p-THF/PDMS surfaces yielded lower *E. coli* adhesion than with PDMS (*p* < 0.0001), while f-CNT/PDMS and f-THF/PDMS surfaces had no effect on cell adhesion (*p* > 0.05). All the composite coatings had the same or lower adhesion than the PDMS control. The coatings that had the lowest cell density after 30 min were those with non-functionalized CNTs (p-samples), while those with functionalized CNTs (f-samples) always presented a higher adhesion (*p* < 0.0001), clearly showing an effect of CNT chemistry on cell adhesion behavior.

From the results, it was also observed that there was a reduction in cell adhesion from p-CNT/PDMS to p-BM/PDMS, as well as from f-CNT/PDMS to f-BM/PDMS (*p* < 0.0001). Since the only difference between them was the milling of the CNTs, this was probably driven by a better degree of dispersion in PDMS achieved for the ball-milled nanotubes, which therefore influenced the distribution of the nanotubes near the interface and possibly the surface roughness of the coatings. On the other hand, using THF to disperse the CNTs also seemed to influence cell adhesion, with an intensification of the response obtained for p-CNT/PDMS and f-CNT/PDMS. This may also suggest that the CNTs in the p-THF/PDMS and f-THF/PDMS surfaces were able to interact more efficiently with the cells, possibly due to their availability at the surface.

Comparing these results with those obtained for the surface energy, it can be noted that for p-CNT/PDMS, f-CNT/PDMS, p-BM/PDMS and f-BM/PMDS surfaces there seemed to be a correlation with the hydrophobic character, where a lower hydrophobicity seemed to favor cell adhesion. These results are in agreement with our previous work, whose findings revealed that even at lower loading values (0.1 wt%), functionalized CNTs could increase cell adhesion by 40% when compared to the PDMS surface with p-CNTs [67]. However, in the p-THF/PDMS and f-THF/PDMS surfaces, an opposite relationship was observed. In fact, other studies have been supporting the theory that hydrophobic surfaces promote cell adhesion [68,69], showing that this relation between hydrophobicity and cell adhesion is not so stringent [70,71]. The fact that these coatings were produced using a different method, which possibly resulted in better dispersion of the CNTs, may have had an influence on their distribution at the surface and thus on the measured surface energy. However, the adhesion values obtained followed the same trend as the remaining groups of surfaces, which suggests that other types of forces and interactions may be driving bacterial adhesion in these composites. It should be noted that in previous works from our group, we have shown that a significant reduction in initial bacterial adhesion may indicate an antifouling behavior in longer biofilm formation assays performed in the hydrodynamic conditions of urinary catheters and stents [46,47].

Although the p-BM/PDMS was the most promising surface, with a reduction in cell adhesion of approximately 60%, p-THF/PDMS composites also showed very encouraging results. Along with the reduction in cell adhesion of about 40%, these surfaces have shown a great improvement in electrical conduction (by seven orders of magnitude when compared to PDMS), which poses advantages in biomedical applications where sensing capabilities are required and similar values of shear stress are applied [72,73,74].

## 3. Materials and Methods

### 3.1. CNT Modification

The original CNT sample was commercially available pristine MWCNTs (Nanocyl^TM^ NC3100, Sambreville, Belgium) produced by catalytic chemical vapor deposition with an average length and diameter of 1.5 µm and 9.5 nm, respectively. p-CNTs were first functionalized by a well-established oxidation treatment with nitric acid (HNO_3_) [49] to produce f-CNTs with oxygenated moieties. Briefly, a sample of p-CNT was oxidized in reflux with HNO_3_ in a Pyrex round-bottom flask containing 300 mL of HNO_3_ 7 M and 3 g of p-CNT, connected to a condenser and the liquid phase was heated at 130 °C with a heating mantle for 180 min. After this process, the f-CNTs were washed with distilled water to neutral pH and dried at 110 °C overnight.

Additionally, both p-CNT and f-CNT samples were mechanically treated by ball-milling (Retsch MM200, Haan, Germany) at 15 vibrations s^−1^ for 180 and 90 min to produce the p-BM and f-BM samples, respectively.

### 3.2. CNT/PDMS Composite Fabrication

The composite materials were fabricated using two different processes—bulk mixing and solution mixing. The first method—bulk mixing—consisted of the direct incorporation of the CNT samples into the PDMS matrix (Sylgard 184 Part A, Dow Corning, Midland, MI, USA; viscosity = 1.1 cm^2^ s^−1^; specific density = 1.03) at 1 wt% CNT loading. Firstly, the CNT samples were dispersed in the PDMS matrix by shear mixing with a magnetic bar at 500 rpm for 30 min, allowing for a rough dispersion of the aggregates. The ball-milling technique enables a better dispersion of the CNT when compared with p-CNT and f-CNT. The CNT/PDMS mixture was then subjected to a sonication procedure (Hielscher UP400S, Teltow, Germany, at 200 Watt and 12 kHz) for at least 60 min until the CNTs were all macroscopically dispersed. However, even after sonication, there were some clusters of CNTs suspended in the composite. After that, a 30 min ultrasound bath (Selecta Ultrasons, Barcelona, Spain) step was added to eliminate the bubbles. The curing agent (Sylgard 184 Part B, Dow Corning) was then added to the base polymer in an A:B proportion of 10:1 and carefully stirred to homogenize the two components without re-introducing bubbles. The composite materials were then deposited as thin layers on top of glass slides by spin coating (Spin150 Polos^TM^, Caribbean, the Netherlands) for 1 min at 6000 rpm for the p-CNT/PDMS and f-CNT/PDMS composites, and at 2000 rpm for the p-BM/PDMS and f-BM/PDMS composites. The former required a higher spin speed since the mixture was more viscous due to the higher degree of aggregation of the CNTs.

The second method used to fabricate the composite materials—solution mixing—consisted of first dispersing the CNTs in THF for about 16 h (resulting in the p-THF and f-THF samples) and then mixing this CNT suspension with the PDMS for additional 6 h at the same 1 wt% CNT loading. Again, the curing agent was added in a 10:1 proportion. The resulting mixture was deposited on the glass slides by manually spreading in such a way to form a uniform layer, and the films were cured in the same conditions as the other materials. The six different materials produced are summarized in Table 3.

### 3.3. Characterization

The four samples of CNTs—p-CNT, f-CNT, p-BM and f-BM—were characterized by N_2_ adsorption isotherms determined at −196 °C with a Quantachrome NOVA 4200e apparatus (Quantachrome Instruments, Boynton Beach, USA). Their textural properties were compared by measuring the (S_BET_) of the various materials. The p-CNT and f-CNT were further characterized by TPD to compare their surface chemistry. The samples were heated from room temperature to 1100 °C at a heating rate of 5 °C min^−1^ with a total flow rate of the helium carrier gas of 25 cm^3^ min^−1^ in an AMI-300 (Altamira Instruments, Pittsburgh, USA) apparatus. About 0.09 g of each sample was analyzed by tracking the m/z signals of 18 (H_2_O), 28 (CO), and 44 (CO_2_) with a Dycor Dymaxion mass spectrometer (Ametek Process Instruments, Pittsburgh, USA). The signals were then processed and analyzed to obtain the total amount of CO and CO_2_ released from the samples. To assess the effect of the incorporation of the CNTs on the thermal stability of the composites, PDMS, p-CNT, p-BM, f-BM and p-THF/composite samples were analyzed by TGA (Netzsch STA 409 PC/PG, Selb, Germany). Samples were heated up to 700 °C at a heating rate of 10 °C min^−1^, under nitrogen flow, and the weight loss monitored to compare the on-set temperature of decomposition among the tested samples.

To obtain an estimative of the surface hydrophobicity of the various composite materials, static CA measurements were performed (Dataphysics Contact Angle System OCA) using the sessile drop technique. Calculations of the surface tension and surface free energy of the solid surfaces (s) were based on the thermodynamic theory [75] by using the Young–Good–Girifalco–Fowkes Equation (1) [76,77], where γ_s_^LW^ and γ_s_^AB^ are the Lifshitz–van der Waals and the Lewis acid-base components of the samples’ surface tension, respectively, and γ_s_^+^ and γ_s_^−^ are the electron acceptor and electron donor parameters, respectively. γ_s_^LW^ and γ_s_^AB^ were obtained by measuring the contact angle (θ) of three different liquids (l) with known surface tension components—water, α-bromonaphthalene, and formamide—followed by the simultaneous resolution of three equations of the type of the Equation (1) where the subscript **s** refers to the surface and the subscript **l** refers to the liquid. The global surface energy (γ_s_^TOT^) was then determined by the sum of these two components using Equation (2):

(1) $$\left(1+cos\theta \right){\gamma_{l}}^{TOT}=2\left(\sqrt{{\gamma_{s}}^{LW}{\gamma_{l}}^{LW}}+\sqrt{{\gamma_{s}}^{+}{\gamma_{l}}^{-}}+\sqrt{{\gamma_{s}}^{-}{\gamma_{l}}^{+}}\right),$$

(2) $${\gamma_{S}}^{TOT}={\gamma_{S}}^{LW}+{\gamma_{S}}^{AB}={\gamma_{S}}^{LW}+2\sqrt{{\gamma_{S}}^{+}{\gamma_{S}}^{-}},$$

From Equations (1) and (2), the hydrophobicity of the surfaces was determined as a measure of the free energy of interaction between two entities of that surfaces immersed in water (*w*)—ΔG_s_^TOT^. According to the thermodynamic theory, a surface is hydrophobic if the interaction between the two entities is stronger than the interaction of each entity with water (∆G_s_^TOT^ < 0 mJ m^−2^) and hydrophilic otherwise (∆G_s_^TOT^ > 0 mJ m^−2^). ΔG_s_^TOT^ was calculated according to Equations (3)–(5), where the surface tension components of the interacting entities are considered:

(3) $$\Delta {G_{s}}^{TOT}=-2{\gamma_{s}}^{TOT}=\Delta {G_{s}}^{LW}+\Delta {G_{s}}^{AB},$$

(4) $$\Delta {G_{s}}^{LW}=-2{\left(\sqrt{{\gamma_{s}}^{LW}}-\sqrt{{\gamma_{w}}^{LW}}\right)}^{2},$$

(5) $$\Delta {G_{s}}^{AB}=-4\left(\sqrt{{\gamma_{S}}^{+}{\gamma_{S}}^{-}}+\sqrt{{\gamma_{W}}^{+}{\gamma_{W}}^{-}}-\sqrt{{\gamma_{S}}^{+}{\gamma_{W}}^{-}}-\sqrt{{\gamma_{W}}^{+}{\gamma_{S}}^{-}}\right),$$

ΔG_s_^LW^ and ΔG_s_^AB^ represent the Lifshitz–van der Waals and the acid-base free energies of cohesion of the surface, respectively [75].

The surface of the various samples was further characterized by SEM. Images of the cross-sections of the coatings were also obtained. SEM analyses were performed using a High-resolution Environmental Scanning Electron Microscope with X-Ray Microanalysis and Electron Backscattered Diffraction analysis—Quanta 400 FEG ESEM (Hillsboro, OR, USA).

Additionally, to assess the effect of the introduction of CNTs in the PDMS matrix on the conductivity properties of the composites, DC electrical conductivity tests were performed. A DC-voltage (V) signal in the range −100–+100 Volt or −10–+10 Volt was applied on each sample produced by the bulk mixing and solution mixing procedure, respectively. The corresponding current (I) was measured at 21 points in 10 runs using two copper contacts that were placed on top of the coating with a separation of 2 cm and then connected to a power source (Keithley Programmable Single Channel DC Power Supplier, Cleveland, OH, USA). The current-voltage curves were obtained and an average value of conductance (I/V) was calculated from a linear fit of the curves.

### 3.4. Cell Cultivation and Harvesting

*E. coli* JM109(DE3) from Promega (Madison, WI, USA) was selected for this study because it has been used in previous works from our group for the evaluation of initial adhesion in antifouling surfaces [67] and because it has was shown to have similar biofilm formation behavior to different clinical isolates, including *E. coli* CECT434 [78]. A starter cell culture was obtained using the same procedure as described in Moreira, et al. [79]. In brief, the cells were collected from a cryo-preserved batch (1 mL aliquots in glycerol stock kept at a constant −80 °C) and thawed at room temperature. Then, 200 mL of culture medium, prepared as previously described [80], were inoculated with 500 μL of cell suspension and incubated overnight at 37 °C with a constant orbital agitation of 120 rpm. A volume of 60 mL of the cultured bacteria was centrifuged (Eppendorf Centrifuge 5810R, Hamburg, Germany) at 3202× *g* for 10 min, resuspended in citrate buffer at pH 5 and centrifuged again in the same conditions. The final cell suspension was then diluted in citrate buffer until an optical density (OD_600 nm_) of 0.1, corresponding to a cell density of 7.6 × 10^7^ cells mL^−1^.

### 3.5. E. coli Adhesion Assays

The bacterial adhesion assays on the coated slides were performed in a PPFC coupled to a system containing a reactor connected to a centrifugal pump and tubing system, which was fed with a steady flow of the *E. coli* suspension. A flow rate of 2 mL s^−1^ was used, which yields a shear rate of 15 s^−1^ and a shear stress of 0.01 Pa [68]. This shear rate is typical for urinary catheters [81] and stents [82], where *E. coli* is one of the most relevant microbial colonizers [45]. The equipment was also coupled to a water bath to keep a constant temperature of 37 °C. For each sample, the medium with *E. coli* was allowed to flow for 30 min, after which the coatings were removed and stained with 4′-6-diamidino-2-phenylindole for later visualization under fluorescence microscopy (Nikon Eclipse LV100 series, magnification 100×, Nikon Corporation, Tokyo, Japan) and total cell counts. The final cell density of each sample was taken into account in the evaluation of bacterial cell adhesion.

### 3.6. Statistical Analysis

Differences between the final cell densities on each surface were tested using a one-way analysis of variance followed by Tukey’s test for pairwise comparisons. Three independent assays were conducted for each surface. Results were considered statistically different when a confidence level >95% was reached (*p* < 0.05). The standard deviation between the three values obtained from the independent experiments is represented by error bars.

## 4. Conclusions

MWCNT/PDMS composite materials with 1 wt% of MWCNTs loading were produced using two different procedures, bulk mixing (p-CNT/PDMS, f-CNT/PDMS, p-BM/PDMS, and f-BM/PDMS) and solution mixing (p-THF/PDMS and f-THF/PDMS). The materials were characterized in terms of surface energy, thermal stability, electrical conductivity and surface topology. Adhesion assays with *E. coli* were performed in an attempt to establish a relationship between the materials’ properties and the tendency for cell adhesion. The results showed that p-CNT were successfully functionalized through oxidation treatment, and p-CNT/PDMS and f-CNT/PDMS had different textural properties, which were reflected in the enhancement of thermal stability of the composites. The conductivity measurements showed a considerable improvement in DC electrical conductivity in the p-THF/PDMS and f-THF/PDMS composites, with possible applications in biosensing devices.

The *E. coli* adhesion assays resulted in reduced adhesion on the composite materials, with the lowest adhesion obtained on the p-BM/PDMS sample, and the highest obtained on the f-THF/PDMS sample. The CA measurements suggested that for the composites produced by bulk mixing (p-CNT/PDMS, f-CNT/PDMS, p-BM/PDMS, and f-BM/PDMS) the adhesion was favored by lower hydrophobicity, while for the p-THF/PDMS and f-THF/PDMS composites the opposite was observed. The p-THF/PDMS sample seemed to be the best compromise between cell adhesion and electrical conductivity.

## Figures and Tables

**Table 1:** Contact angle results and the calculated values of the surface energy of the samples.

Sample	θ_w_	θ_br_	θ_form_	γ_s_^−^	ΔG_s_^LW^	ΔG_s_^AB^	ΔG_s_^TOT^
PDMS	113.6° ± 0.6	87.6° ± 1.8	111.2° ± 0.6	4.5 ± 0.9	−2.9 ± 0.5	−58.9 ± 4.3	−61.8 ± 4.4
p-CNT/PDMS	117.0° ± 0.7	80.4° ± 0.7	110.4° ± 0.8	2.5 ± 0.4	−1.2 ± 0.1	−70.2 ± 2.9	−71.4 ± 2.9
f-CNT/PDMS	116.6° ± 0.6	76.6° ± 1.5	111.1° ± 0.5	3.0 ± 0.6	−0.6 ± 0.2	−67.0 ± 3.7	−67.7 ± 3.7
p-BM/PDMS	121.5° ± 0.3	88.8° ± 0.9	115.7° ± 0.4	1.9 ± 0.3	−3.2 ± 0.3	−73.8 ± 2.2	−77.1 ± 2.2
f-BM/PDMS	113.6° ± 0.4	71.3° ± 1.4	109.1° ± 0.6	4.0 ± 0.7	−0.1 ± 0.1	−61.5 ± 3.3	−61.7 ± 3.3
p-THF/PDMS	116.6° ± 0.3	80.5° ± 1.7	112.0° ± 0.5	3.2 ± 0.7	−1.2 ± 0.3	−65.7 ± 3.8	−67.0 ± 3.8
f-THF/PDMS	125.6° ± 0.4	92.3° ± 1.5	118.6° ± 0.9	1.2 ± 0.4	−4.3 ± 0.5	−80.1 ± 3.7	−84.5 ± 3.8

θ_w_, θ_br_ and θ_form_: the average of measured contact angles with water, α-bromonaphthalene and formamide, respectively; γ_s_^−^: electron donor parameter; ΔGs^LW^ and ΔG_s_^AB^: Lifshitz–van der Waals and acid-base free energies of the surface, respectively; ΔG_s_^TOT^: free energy of interaction between two entities of the surfaces immersed in water. Values of γ_s_^−^, ΔG_s_^LW^, ΔG_s_^AB^ and ΔG_s_^TOT^ are in mJ m^−2^.

**Table 2:** Direct current mean conductance of the different composite coatings measured in a specified voltage interval (−100–100 Volt, for the glass, PDMS, p-CNT/PDMS, f-CNT/PDMS, p-BM/PDMS, and f-BM/PDMS samples, and −10–10 Volt for the p-THF/PDMS and f-THF/PDMS samples).

Sample	Conductance (S)
PDMS	1.9·10^−12^ ± 9.9·10^−14^
p-CNT/PDMS	1.9·10^−12^ ± 5.8·10^−14^
f-CNT/PDMS	1.3·10^−12^ ± 5.0·10^−14^
p-BM/PDMS	1.3·10^−12^ ± 5.8·10^−14^
f-BM/PDMS	1.4·10^−12^ ± 6.0·10^−14^
p-THF/PDMS	1.5·10^−5^ ± 4.2·10^−7^
f-THF/PDMS	6.9·10^−7^ ± 2.0·10^−8^

**Table 3:** Description of the materials prepared through the bulk mixing and solution mixing processes.

Material	CNT Treatment	Method
p-CNT/PDMS	none	bulk mixing
f-CNT/PDMS	oxidation with nitric acid	bulk mixing
p-BM/PDMS	ball-milling	bulk mixing
f-BM/PDMS	oxidation with nitric acid; ball-milling	bulk mixing
p-THF/PDMS	none	solution mixing
f-THF/PDMS	oxidation with nitric acid	solution mixing
